# Phylogeny and Functional Differentiation of the Terpene Synthase Gene Family in Angiosperms with Emphasis on *Rosa chinensis*

**DOI:** 10.3390/ijms26052113

**Published:** 2025-02-27

**Authors:** Qi Li, Yifang Peng, Tao Zhao, Qijing Dong, Qian Yang, Xiaoyu Liu, Yu Han

**Affiliations:** 1 Beijing Key Laboratory of Ornamental Plants Germplasm Innovation & Molecular Breeding, Beijing Laboratory of Urban and Rural Ecological Environment, Key Laboratory of Genetics and Breeding in Forest Trees and Ornamental Plants, Ministry of Education, State Key Laboratory of Efficient Production of Forest Resources, National Engineering Research Center for Floriculture, School of Landscape Architecture, Beijing Forestry University, Beijing 100083, China; 2 State Key Laboratory of Crop Stress Biology for Arid Areas, Shaanxi Key Laboratory of Apple, College of Horticulture, Northwest A&F University, Yangling 712100, China

**Keywords:** genome wide identification, synteny analysis and networks, TPS, super rosids, bifunctional gene

## Abstract

Terpenes are pivotal for plant growth, development, and adaptation to environmental stresses. With the advent of extensive genomic data and sophisticated bioinformatics tools, new insights into the evolutionary dynamics and functional diversification of terpene synthases (TPSs) have emerged. Despite genome-wide identifications of the TPS family in certain species, comprehensive cross-species analyses remain scarce. In this study, we conducted a genome-wide identification and subgroup classification of TPS families across 115 angiosperms with available genomic sequences. Our phylogenomic synteny network analysis elucidated the complex evolutionary history of TPS genes, revealing notable expansions and contractions among subgroups. Specifically, TPS-a showed significant expansion, while TPS-b was variably lost in some Poaceae, indicating adaptive responses. TPS-c maintained considerable conservation across species, whereas TPS-e/f diverged into distinct evolutionary trajectories despite functional overlap, with TPS-e further splitting into two angiosperm-specific clades. The TPS-g subgroup displayed lineage-restricted distribution, primarily in super-rosids and monocots. Notably, TPS-d and TPS-h subgroups were absent in angiosperms. Employing *Rosa chinensis* as a case study, we identified RcTPS23, a conserved bifunctional terpene synthase, highlighting the utility of cross-species synteny data in functional prediction. This comprehensive analysis elucidates the phylogenetic and functional landscape of TPS subgroups in angiosperms, providing a robust framework for predicting TPS function and guiding further functional investigations.

## 1. Introduction

Terpenoids constitute a vast group of natural compounds, with over 50,000 distinct types identified across a broad spectrum of organisms including plants, bacteria, and invertebrates [1,2,3]. These compounds are primarily composed of concatenated C_5_ isoprenoid units, with structural variations such as monoterpenes (C_10_), sesquiterpenes (C_15_), and diterpenes (C_20_) defined by the number of these isoprenoid blocks [3,4]. The biosynthesis of terpenoids is facilitated through two principal pathways: the mevalonate (MVA) pathway and the methyl-erythritol phosphate (MEP) pathway, both of which produce the universal precursors, isopentenyl diphosphate (IPP) and dimethylallyl diphosphate (DMAPP) [5]. In plants, a large family of structurally related enzymes known as terpene synthases/cyclases (TPSs) orchestrates terpenoid biosynthesis. Typically, TPSs in plastids catalyze the cyclization of substrates such as geranyl/neryl diphosphate (GPP/NPP, C_10_; neryl being the cis-isomer of geranyl) to yield monoterpenes, or convert geranylgeranyl/nerylneryl diphosphate (GGPP/NNPP, C_20_) into diterpenes. Conversely, cytosolic TPSs primarily catalyze the transformation of trans/cis-farnesyl diphosphate (*E*, *E*-FPP/*Z*, *Z*-FPP, C_15_) into sesquiterpenes [6,7,8]. However, the specific localization of terpene biosynthesis can vary, with instances of monoterpenes and diterpenes also being synthesized in the cytosol, and sesquiterpenes in the plastids [9,10,11]. Given the crucial role of terpenes in plant biology, research into TPSs has been extensive.

The protein sequence of TPS is characterized by two domains: the N-terminal domain (Pfam ID PF01397) includes the ‘RRX_8_W’ (R, arginine; W, tryptophan; X, alternative amino acid) motif, and the C-terminal domain (Pfam ID PF03936) provides binding sites for Mg^2+^ and Mn^2+^ [4,12]. The C-terminal domain is further divided into α, β, and γ domains. The α domain is a highly α-helical “isoprenoid” or farnesyl diphosphate synthase (FPPS or FPPase) fold, with catalysis mediated via ‘DDxxD’ motifs using a Mg^2+^-dependent “ionization-initiated” mechanism [13]. The β and γ domains are located between a pair of α-helical double-barrel domains and carry out a “protonation-initiated” mechanism utilizing a catalytic ‘DxDD’ motif [8]. The taxonomy, distribution, and products of TPSs in plants have been well studied (Table 1) [12,13,14,15,16,17,18].

The TPS family is mid-sized and highly diversified in plants. The ancestral TPS was likely a bifunctional Class I and II diterpene synthase producing the ent-kaurene required for phytohormone production [19]. The ancestral TPS genes underwent gene duplication at least twice, leading to three ancient TPS lineages: TPS-c, TPS-e/f, and the remaining TPS (h/d/a/b/g) subfamilies (also called subgroups) [8]. TPS-a is the largest subgroup and expanded after the split of the monocot and dicot lineages [20]. The TPS-c subfamily enzymes CPS (copalyl diphosphate synthase) and KS (ent-kaurene synthase) catalyze the committed steps in gibberellin biosynthesis, which governs stem elongation, seed germination, and flowering time across land plants [19]. The evolutionary characteristics of the other subgroups in complex angiosperms have not yet been fully elucidated.

TPS families have been extensively studied in diverse plant species, including *Arabidopsis thaliana* (L.) Heynh. [21], tomato (*Solanum lycopersicum* L.) [8,16], grapevine (*Vitis vinifera* L.) [22], *Eucalyptus grandis* W. Hill [23], apple (*Malus domestica* Borkh.) [24], *Mentha longifolia* (L.) Huds. [25], common wheat (*Triticum aestivum* L.) [26], and Orchidaceae [27], etc. Both transcriptome-based analysis and the isolation of individual genes have contributed to the identification of increasing numbers of plant *TPS* genes [28,29]. The increasing availability of high-quality genomic resources means that it should now be possible to comprehensively analyze the evolution and functional diversification of *TPSs*. We reasoned that building networks of the phylogenetic and syntenic relationships of *TPSs* across numerous species could provide insights into the evolution of these important genes, which exhibit variation and conservation in both gene sequence and biochemical function.

Monoterpene alcohols and other volatile terpene substances provide roses (*Rosa* spp.) with their scent [30,31,32], which is an important target for rose breeders. As a eudicot species within the super-rosids clade—a major evolutionary radiation of core dicots—rose exhibits genomic features characteristic of this advanced angiosperm group [33]. This phylogenetic context helps explain the observed expansion of specialized metabolic gene families like TPSs. In addition, highly volatile terpene compounds are widely used in essential oil production [34]. A unique monoterpene biosynthesis enzyme in rose, *Rosa hybrida* nudix hydrolase 1 (RhNUDX1), shows geranyl diphosphate diphosphohydrolase activity in vitro and is involved in geraniol biosynthesis [4]. However, little is known about rose TPSs. A high-quality genome sequence is now available for *R. chinensis* ‘Old Blush’: (1) Its status as a diploid progenitor (2n = 2x = 14) of modern tetraploid garden roses simplifies genomic analyses; (2) The cultivar’s exceptional terpene volatile diversity provides a rich phenotypic trait for gene-function correlation; (3) As a founder genotype in rose breeding programs, functional insights gained here can directly inform fragrance enhancement strategies [33]. The availability of ‘Old Blush’ genome sequence provides an opportunity to explore the relationships between TPSs and thereby lay the foundation for analysis of terpene-related processes in rose, such as fragrance formation, plant–insect interaction, and phytohormone biosynthesis.

In this study, systematic characterization across diverse angiosperms were used effective methods: (1) Profile HMM searches (HMMER3.0) provide superior sensitivity for detecting divergent TPS homologs; (2) Dual-domain verification (PF01397/PF03936) ensures accurate family membership assignment; (3) Stringent *E*-value thresholds (<10^−3^) balance discovery rates with false positives in large-scale analyses. We performed genome-wide identification and subgroup classification of the TPS families in 115 angiosperms with sequenced genomes (including super-asterids, super rosids, basal-eudicots, monocots, *Liriodendron chinense* (Hemsl.) Sarg. and *Amborella trichopoda* Baill.) and constructed a phylogenomic synteny network to investigate their evolutionary traits. We then used *R. chinensis* as an example species and performed a detailed comparative analysis of TPS subfamily members. Based on synteny analysis, we discovered and validated a bifunctional protein, RcTPS23, which has both linalool and nerolidol synthase activity. Our findings further expand our understanding of the phylogeny and functional differentiation of this important gene family in angiosperms and pave the way for analysis of functional diversity and conservation of TPSs from a new perspective.

## 2. Results

### 2.1. Genome-Wide Identification and Subgroup Classification of TPSs in 115 Angiosperms

**Figure 1:**
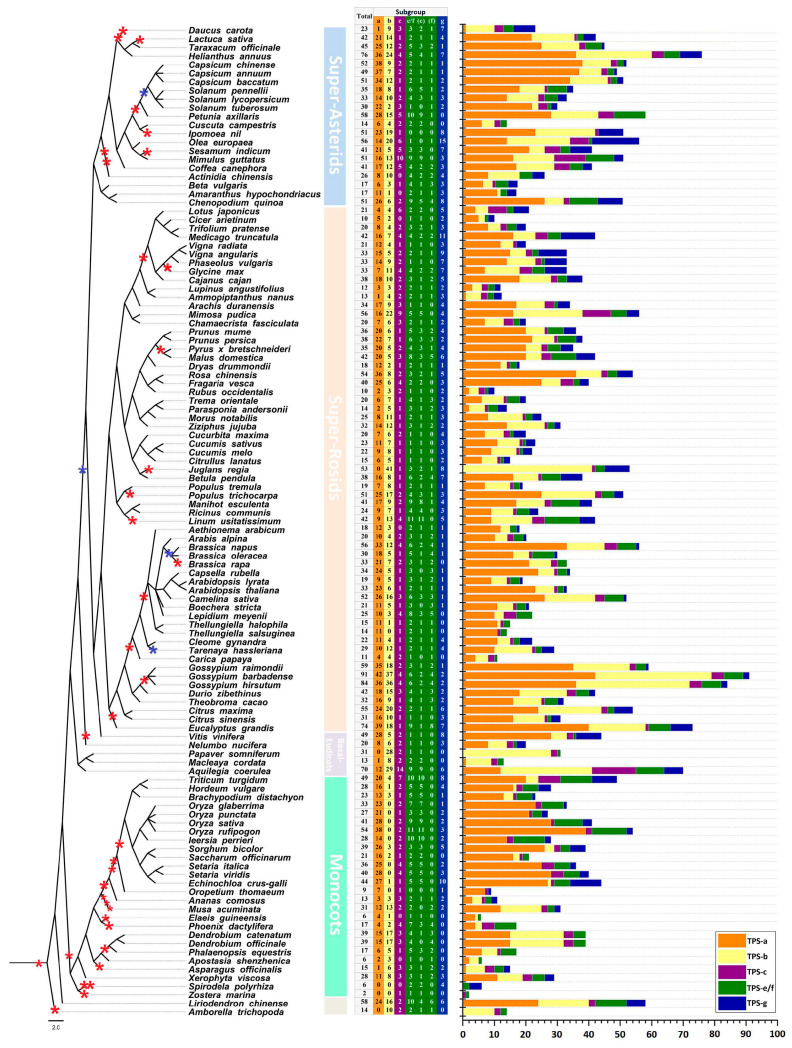
Phylogenetic distribution and genomic organization of TPS genes in angiosperms. (**Left**) Phylogenetic tree of 115 angiosperm species. Major clades are color-coded: super-asterids (light blue), super-rosids (light orange), basal eudicots (light purple), and monocots (light green). *Liriodendron chinense* (magnoliids) and *Amborella trichopoda* (basal angiosperm) are highlighted in light gray. Red asterisks mark whole-genome duplication (WGD) events, blue asterisks indicate whole-genome triplication (WGT) events based on published genomic data [35,36]. (**Center**) The number of TPS genes identified per species, with color gradients indicating subgroup composition: TPS-a (orange), TPS-b (yellow), TPS-c (purple), TPS-e/f (green), TPS-g (blue). Complete data in Table S1. (**Right**) Comparative distribution of TPS subgroups gene counts across species. With column lengths representing subgroup member counts and color scheme corresponds to TPS subgroups as in center panel.

To gain a comprehensive understanding of TPSs in angiosperms, we chose 115 angiosperms with available genomic sequences to construct a phylogenetic tree and used published genomes data to identify TPS sequences (Figure 1). Our genome-wide sequence homology search, focused on two conserved domains (PF01397 and PF03936), identified 3802 TPS genes (Table S1). The number of TPS genes varied significantly among the species studied, ranging from 91 in *Gossypium barbadense* L. to just two in *Zostera marina* L. Notably, five monocot species, including *Oropetium thomaeum* (L.f.) Trin. and *Elaeis guineensis* Jacq., harbored fewer than ten TPS genes each.

Based on conserved domain motif and phylogenetic analyses, we further divided the 3802 TPSs into five subgroups: TPS-a, TPS-b, TPS-c, TPS-g, and TPS-e/f; no TPS-d and TPS-h subgroup were identified (Table S2). We arranged the subgroups based on the phylogenetic order of the 115 angiosperms, as shown in Figure 1. TPS-a was the largest subgroup, with 1806 members, followed by TPS-b. For most (85 out of 115) angiosperms, the TPS-a subgroup accounts for the largest proportion of TPS families. The length of a branch in a phylogenetic tree has a certain relationship with the evolutionary times of the corresponding species [5,20,21]. We observed a significant trend where species positioned at the termini of longer evolutionary branches exhibit an expanded number of members in the TPS-a subgroup, such as the Poaceae branch in monocots (from *Triticum turgidum* L. to *Echinochloa crus-galli* (L.) P.Beauv.), the Solanaceae branch in super-asterids (from *Capsicum chinense* Jacq. to *Petunia axillaris* (Lam.) Britton, Sterns & Poggenb.), and the Rosaceae branch in super-rosids (from *Prunus mume* (Siebold) Siebold & Zucc. to *Fragaria vesca* L.) (orange bars in Figure 1). Other TPS subgroups did not exhibit such a pattern, especially TPS-b, which was the second largest subgroup. In the Poales branch, the size of TPS-b subgroup did not exceed four, and eight Poaceae plants lack TPS-b subgroup members (yellow bars in Figure 1). Our data also revealed the absence of some subgroups in various species. For example, the TPS-a, -b, and -f subgroups were not observed in the aquatic plants *Z. marina* and *Spirodela polyrhiza* (L.) Schleid. In addition, five basal-eudicots lack TPS-f subgroup members. The TPS-c and TPS-e/f subgroups contain TPSs involved in ent-kaurene production, which is required for primary metabolism [19]. We found that no angiosperm had lost both TPS-c and TPS-e/f subfamily members. In most angiosperms of our study, the expansion of TPS gene families was due to the expansion of the TPS-a and TPS-b subgroups, whereas the quantities of TPSs in TPS-c, TPS-e/f, and TPS-g subgroups had not fluctuated greatly.

### 2.2. Synteny Analysis of TPS Gene Families Across 115 Plant Genomes

To elucidate the evolutionary conservation and diversification of terpene synthase (TPS) genes, we constructed synteny networks for 115 plant genomes. Synteny networks are crucial for depicting homologous relationships between gene pairs and tracing the evolutionary paths of genes [37,38]. In these networks, nodes represent TPS genes with syntenic relationships, while edges denote the evolutionary lineages connecting these genes. Our analysis identified 22,035 edges and 1618 nodes, which we organized into 127 distinct communities or node clusters (Tables S3–S5). These clusters were subsequently mapped using phylogenetic profiling, which visually represents the presence or absence of specific syntenic TPS clusters across the 115 angiosperms (Figure S1, Table S6). Notably, Figure S1 reveals the presence of species-specific TPS clusters within particular families, such as Solanaceae, Brassicaceae, and Poaceae, suggesting these clusters may have originated from whole-genome duplication events. Conversely, the absence of specific TPS clusters in some clades may be attributed to the limited number of species examined, underscoring potential gaps in our current genomic coverage.

**Figure 2:**
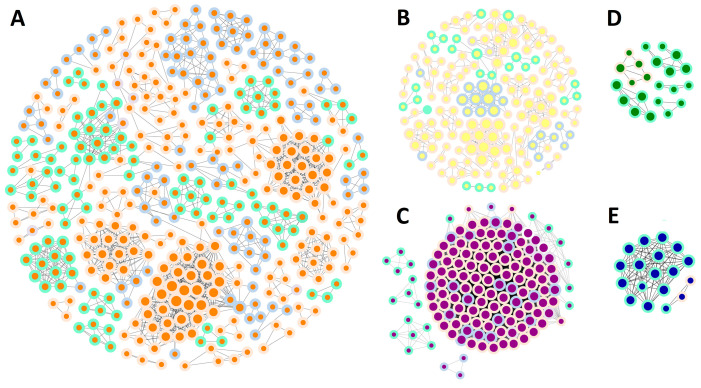
Syntenic network analysis of TPS gene family evolution. (**A**–**E**) Circos plots showing syntenic relationships within TPS subgroups: (**A**) TPS-a (orange), (**B**) TPS-b (yellow), (**C**) TPS-c (purple), (**D**) TPS-e/f (green), (**E**) TPS-g (blue). Each node represents a TPS gene, with inner ring color indicating subgroup and outer ring color denoting species group (super-asterids: light blue; super-rosids: light orange; basal eudicots: light purple; monocots: light green; *L. chinense* and *A. trichopoda*: light gray). Node sizes reflect the number of synteny connections per node, with detailed col-linear TPS gene pair information available in Table S5.

Syntenic relationships among species are invaluable for evolutionary analysis, providing insights into gene conservation and divergence over time [39]. To further uncover the potential evolutionary stories of TPSs in angiosperms, we constructed five subgroup-specific networks whose nodes represent subgroups TPS-a, -b, -c, -e/f, and -g (Figure 2A–E, Table S5). Each subgroup showed a specific synteny pattern, and the synteny patterns of certain subgroups were unique to specific plants [40]. The TPS-a network was the largest group, with 71 clusters and nodes that were widespread across angiosperms. A similar pattern emerged for the TPS-b network: some small clusters had the same outer circle color (species group), with interconnected nodes within the same species categories (Figure 2A,B). As shown in Figure 2C, the TPS-c network was highly interconnected, with nodes belonging to super-asterids, super-rosids, and basal eudicots and several nodes from monocots distributed in the periphery. Several nodes from super-asterids and monocots form four small clusters. Both the TPS-e/f and TPS-g networks were small and distributed in super-rosids and monocot species. The synteny pattern of TPS-c was different from the other subgroups. The tight syntenic junctions of TPS-c indicated that this subgroup was highly conserved within eudicots.

### 2.3. Phylogenetic Analysis of the TPS-c and TPS-e/f Subgroups

**Figure 3:**
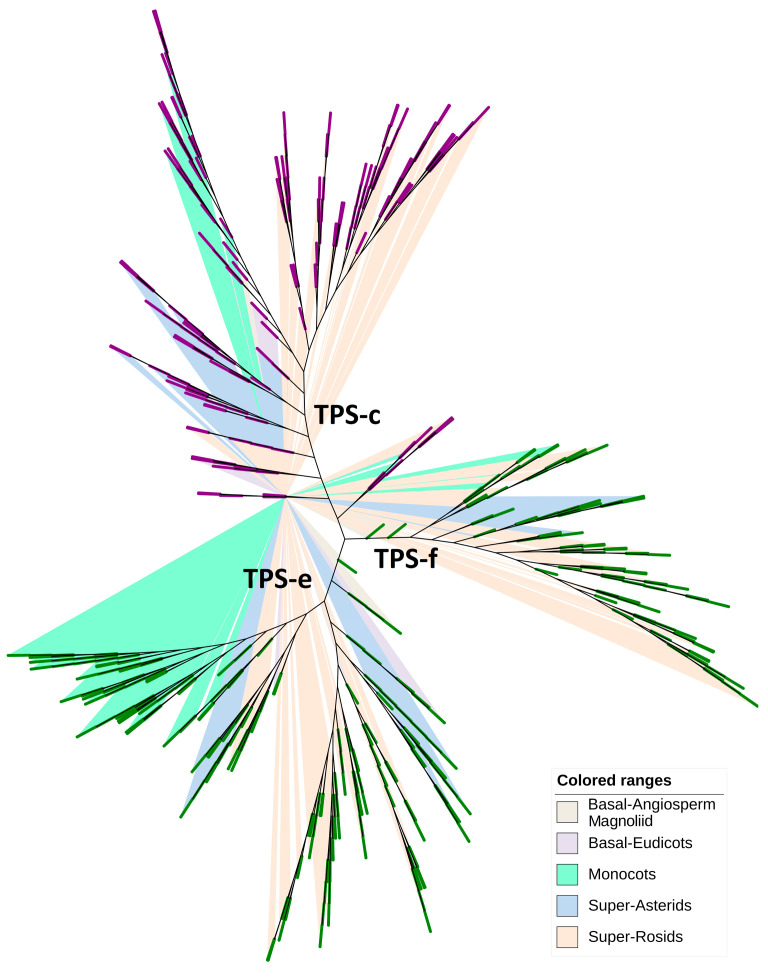
Phylogenetic relationships of TPS-c and TPS-e/f subgroups across 115 angiosperm species. The TPS-c clades are shown in purple, and the TPS-e/f clades are shown in green. The phylogenetic tree was constructed using the TPS-c and TPS-e/f members from 115 angiosperms (Table S1). The clades are highlighted in different colors: super-rosids (light orange), monocots (light green), super-asterids (light blue), basal-eudicots (light purple), basal-angiosperms and magnoliids (light gray).

To elucidate the phylogenetic relationships and functional diversification within the TPS-c and TPS-e/f subgroups, conserved across the angiosperms yet demonstrating remarkable functional diversity, we employed a robust analytical framework. By conducting a comprehensive multiple sequence alignment of TPS genes from 115 angiosperm species, we constructed maximum-likelihood phylogenetic trees to dissect their evolutionary trajectories. The phylogenetic delineation, presented in Figure 3, Table S2, and Figure S2, clearly segmented these genes into three distinct clades: TPS-c, TPS-e, and TPS-f.

Our results reveal a stable representation of TPS-c genes across diverse plant lineages, encompassing 21 super-asterids, 61 super-rosids, five basal-eudicots, 26 monocots, one basal-angiosperm, and one magnoliid. Conversely, the TPS-e and TPS-f subgroups, traditionally considered a single entity due to overlapping functional attributes and structural homologies, exhibited discernible phylogenetic bifurcation. Notably, TPS-e diverged into two subclades: one enriched with monocot genes, indicative of an expansion specific to this clade post the monocot-dicot evolutionary split, while the other was devoid of them. These findings accentuate the intricate evolutionary dynamics of the TPS gene family, reflecting both the retention of ancestral traits and the diversification essential for ecological adaptations.

### 2.4. Comparative Analysis of the TPS Family in R. chinensis

**Figure 4:**
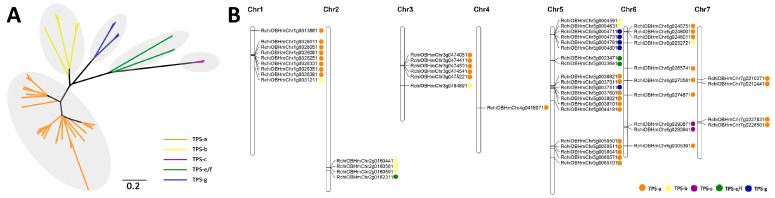
Phylogenetic and chromosomal distribution of RcTPSs in *R. chinensis*. (**A**) This panel shows a phylogenetic tree of TPS proteins from *R. chinensis*, constructed using the maximum-likelihood method with 1000 bootstrap replicates via RAxML. The tree segregates TPS proteins into six subgroups, each indicated by a distinct color. The composition and relationships of these subgroups are detailed in Table S7. (**B**) Chromosomal distribution of putative functional terpene synthase genes across the chromosomes (denoted as Chr) of *R. chinensis*. Each gene is represented by a small dot, color-coded by subfamily: TPS-a (orange), TPS-b (yellow), TPS-c (purple), TPS-e/f (green), and TPS-g (blue), providing a visual representation of gene location and subgroup prevalence.

Among the 12 Rosales species examined, *R. chinensis* hosts the most extensive TPS gene family. We constructed a phylogenetic tree of 54 RcTPS genes, which grouped into three main clades as depicted in Figure 4A. The predominant clade comprises 36 genes from the TPS-a subgroup, significantly larger than the others, which include eight TPS-b and five TPS-g members in the second clade, and two TPS-c and three TPS-e/f members in the third clade. Detailed phylogenetic analysis revealed two sub-clades within the TPS-a subgroup, characterized by notably diverse branch lengths, indicating a significant expansion relative to other subgroups (Figure 4A). We further explored the chromosomal distribution of these 54 RcTPS genes, finding a pronounced uneven distribution across the seven chromosomes of *R. chinensis* (Figure 4B). Notably, chromosome 5 (Chr5) harbors 20 RcTPS genes, whereas chromosome 4 (Chr4) contains only one. All members of the TPS-g and TPS-c subfamilies are localized to Chr5 and Chr6, respectively. Except for Chr2, TPS-a gene clusters are present on six chromosomes. This genomic architecture, combined with our phylogenetic findings, underscores the expansion of the TPS-a genes. Additionally, gene structure analysis provides further evolutionary insights; for instance, most TPS-a, TPS-b, and TPS-g subgroup members feature seven exons, while TPS-c and TPS-e/f subgroup members typically have 13 to 15 exons (Figure S3). These structural patterns, similar to those observed in *A. thaliana*, *E. grandis*, and *M. domestica* [21,23,41], suggest a relative conservation of exon features across these species.

**Figure 5:**
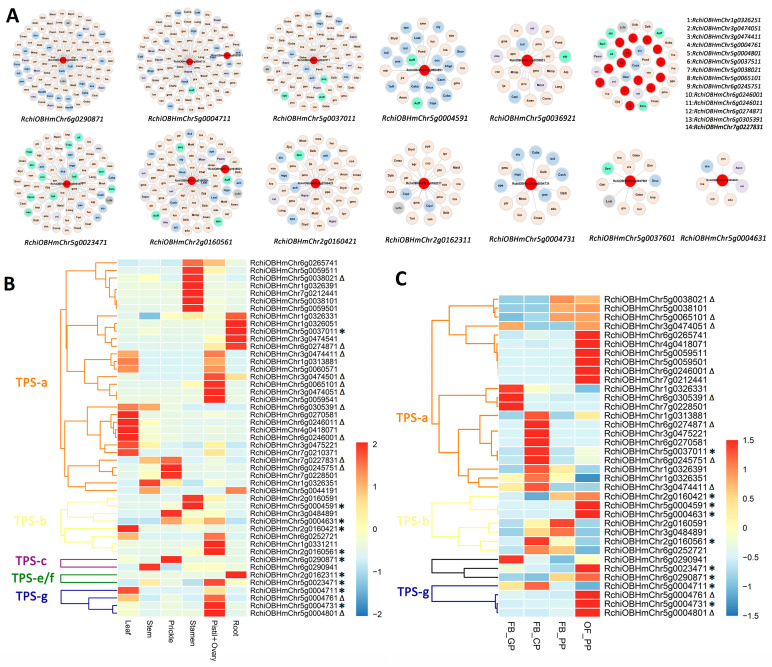
Synteny networks and spatiotemporal expression patterns of RcTPSs in *R. chinensis*. (**A**) Synteny clusters of TPSs across various species with *R. chinensis* highlighted. Each node represents one TPS, color-coded to indicate species classification: super-asterids (light blue), super-rosids (light orange), basal eudicots (light purple), monocots (light green), *L. chinense* and *A. trichopoda* (light gray). Nodes specific to *R. chinensis* are marked in red, with gene names labeled beneath each cluster. (**B**) Spatial expression profiles of *TPS* genes across various tissues of ‘Old Blush’ (root, leaf, stamen, pistil, ovary, stem, and prickle), using RNA-seq data sourced from NCBI (accession number PRJNA546486). Black triangles indicate RcTPSs with a single synteny connection; black asterisks denote genes with multiple synteny connections. (**C**) Temporal expression patterns of *TPS* genes during four developmental stages of rose petals in ‘Old Blush’: FB_GP (green petals in a flower bud), FB_CP (petals changing color in a flower bud), FB_PP (pink petals in a flower bud), and OF_PP (pink petals in an open flower), based on RNA-seq data from NCBI (accession number PRJNA351281). Symbols are used consistently as in (**B**) to indicate the connectivity of synteny edges among the RcTPSs.

We explored the syntenic relationships of 54 identified RcTPSs across 115 angiosperms, finding that 27 of these genes are syntenic homologs with TPSs from other species. The relationships involving RcTPSs were individually illustrated, with each RcTPS positioned centrally in the diagrams (Figure 5A). Notably, two RcTPSs (RchiOBHmChr5g0023471 and RchiOBHmChr2g0160561) demonstrated extensive syntenic connections. The majority of RcTPSs (23 out of 27) predominantly showed synteny with TPSs from species within the super-rosid and super-asterid groups. Moreover, two RcTPSs (RchiOBHmChr1g0326251 and RchiOBHmChr5g0004801) appeared as Rosaceae-specific syntelogs, indicating unique syntenic relationships confined to this family. Additionally, three distinct pairs of syntenic genes were identified between *R. chinensis* and *V. vinifera*, highlighting specific evolutionary links (items 9, 10, and 11 in Figure 5A).

We analyzed the gene expression patterns of *RcTPSs* using RNA-seq data from various rose tissues and across four critical developmental stages of petals. From two distinct RNA-seq datasets, 47 and 36 *RcTPSs* were identified, as detailed in Tables S8 and S9. The expression patterns and synteny relationships for each subgroup are illustrated in Figure 5B,C. Predominantly, *RcTPSs* exhibited heightened expression levels in specific tissues, with several genes presenting multiple synteny edges (marked by black asterisks), particularly within subgroups TPS-b, TPS-c, TPS-e/f, and TPS-g. A notable observation was that only one *RcTPS* (*RchiOBHmChr5g0037011*) from the TPS-a subgroup demonstrated syntenic relationships with more than one species and showed expression across various rose tissues. Additionally, eleven and eight RcTPSs from the TPS-a subgroup exhibited a single synteny edge with genes from other species, as shown in Figure 5B,C. Petals are the main tissues from which fragrance is released in rose, and many terpenes related to flower fragrance are mainly synthesized in petals. As the rose flower opens, the amount of fragrance released increases [42]. Each subgroup contains members that were highly expressed in OF_PP stage petals. These finding lay the foundation for further in-depth study of RcTPS function.

### 2.5. Identification of the Bifunctional Terpene Synthase RcTPS23

**Figure 6:**
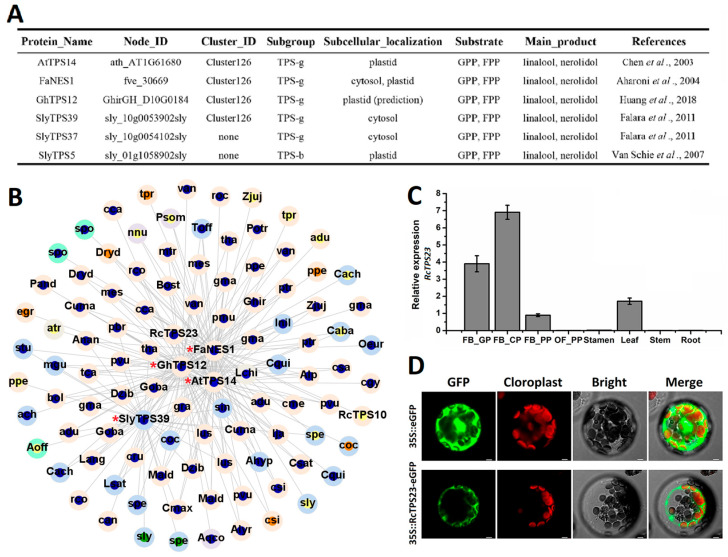
Syntenic localization, spatiotemporal expression, and subcellular localization of RcTPS23. (**A**) Catalog of TPS enzymes capable of catalyzing the substrates GPP and FPP to produce linalool or nerolidol [16,43,44,45,46]. (**B**) Syntenic analysis of key TPSs, including AtTPS14, FaNES1, GhTPS12, and SlyTPS39 (highlighted with red asterisks). Each node is annotated with species abbreviations and color-coded: nodes in the outer loop represent different plant groups—super-asterids (light blue), super-rosids (light orange), basal eudicots (light purple), monocots (light green), *L. chinense* and *A. trichopoda* (light gray); nodes in the inner loop represent TPS subgroups, coded as TPS-a (orange), TPS-b (yellow), TPS-c (purple), TPS-e/f (green), and TPS-g (blue). (**C**) Expression analysis of *RcTPS23* across four developmental stages of rose petals and various tissues, conducted via qRT-PCR: FB_GP (green petals in a flower bud), FB_CP (petals changing color in a flower bud), FB_PP (pink petals in a flower bud), and OF_PP (pink petals in an open flower). Expression data are expressed as mean ± standard deviation from three biological replicates. (**D**) Subcellular localization of 35S::RcTPS23-eGFP fusion protein (green) in *A. thaliana* protoplasts. Using 35S::eGFP as the control. Red fluorescence corresponds to chloroplast autofluorescence detected under 488 nm excitation. Scale bars = 10 μm.

TPS functions are often related to their subcellular localization (plastid or cytosol), and some TPSs can react with both GPP and FPP when expressed in vitro. The most common of these potentially bifunctional TPSs, whose products are monoterpene linalool and sesquiterpene nerolidol, have been reported in *A. thaliana*, strawberry, tomato, cotton, and other species (Figure 6A) [16,43,44,45,46]. BLAST (version 2.13.0) analysis of the protein sequences in our data revealed that most of these TPSs belong to the TPS-g subgroup and synnet Cluster126. We constructed a syntenic network of AtTPS14, FaNES1, GhTPS12, and SlyTPS39 and found that most nodes were in subgroup TPS-g and belong to various species (Figure 6B). Two rose TPSs were present in this syntenic network. We cloned these genes and named them *RcTPS23* (*RchiOBHmChr5g0004711*) and *RcTPS10* (*RchiOBHmChr2g0160421*). The sequence of *RcTPS10* contained a frameshift. *RcTPS23* reached its highest expression levels in stage FB_CP petals but was expressed at very low levels in stamens, stems, roots, and OF_PP stage petals (Figure 6C). We investigated the subcellular localization of RcTPS23 by expressing RcTPS23-eGFP fusion protein in *A. thaliana* protoplasts (Figure 6D). Confocal laser scanning microscopy revealed that RcTPS23-eGFP localized to the cytosol, suggesting that *RcTPS23* might function in sesquiterpene biosynthesis.

**Figure 7:**
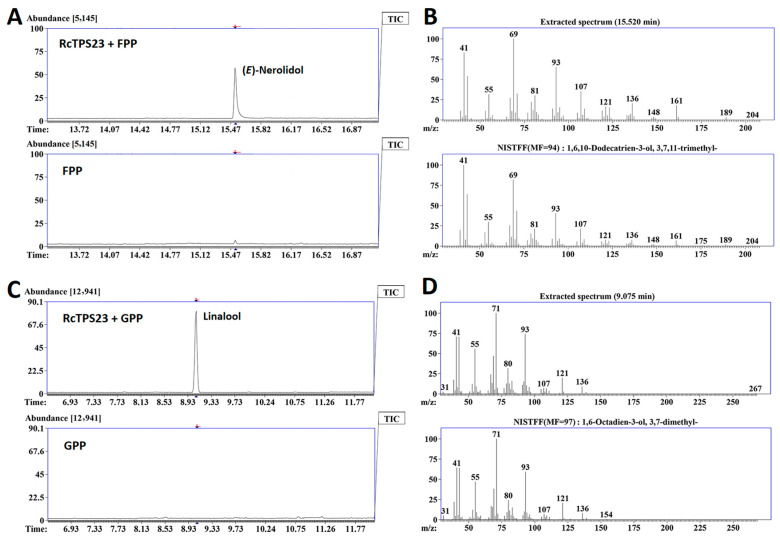
Dual catalytic activity of RcTPS23 in producing (*E*)-nerolidol and linalool in vitro. (**A**) Gas chromatography-mass spectrometry (GC-MS) chromatograms displaying sesquiterpene synthesis by recombinant RcTPS23 using farnesyl diphosphate (FPP) as a substrate (top panel). The blank control, containing only the substrate FPP, is shown for comparison. (**B**) Mass spectrum of (*E*)-nerolidol synthesized by incubating FPP with purified RcTPS23 protein (top panel), compared against the mass spectrum of (*E*)-nerolidol from the NIST standard reference database (bottom panel). (**C**) GC-MS chromatograms illustrating monoterpene production by recombinant RcTPS23 with geranyl diphosphate (GPP) as the substrate (top panel). The corresponding blank control includes only the substrate GPP. (**D**) Mass spectrum of linalool produced following incubation of GPP with purified RcTPS23 protein (top panel). For comparative analysis, the bottom panel displays the mass spectrum of linalool from the NIST standard reference database.

Finally, to perform functional analysis, we heterologous expressed *RcTPS23* in *Escherichia coli* strain BL21 (DE3) and analyzed the in vitro chemical products of the resulting recombinant proteins using different substrates by GC-MS. The product produced by RcTPS23 and the substrate FPP was (*E*)-nerolidol (Figure 7A,B). However, linalool was produced when GPP was used as the substrate (Figure 7C,D). In these assays, linalool and nerolidol were identified by comparing mass spectra. Each reaction had a blank control and three biological replications. In summary, we used the syntenic relationships of TPSs to identify the TPS-g subgroup member *RcTPS23* and analyzed its gene expression pattern in rose. We determined that RcTPS23 was a bifunctional terpene synthase that produces nerolidol and linalool in vitro and might possess nerolidol biosynthetic activity in vivo due to its localization in the cytoplasm.

## 3. Discussion

The TPS families in the plant kingdom are highly diversified [20]. In this study, we performed the first large-scale assessment of the number of TPS-subgroup members in 115 angiosperms (Tables S1 and S2). Despite the key roles that whole-genome duplication (WGD) and whole-genome triplication (WGT) events often have in increasing gene family size [47], we did not find evidence for such effects on TPS families (Figure 1). A previous study suggested that the large-scale expansion of the TPS family occurred after the divergence of dicot and monocot plants [48]. Our data support this conclusion. Moreover, we determined that the expansion of the TPS family was mainly due to the expansion of the TPS-a and TPS-b subgroups. The expansion of TPS-a suggests a correlation between the rate of evolutionary change—reflected by longer branch lengths—and the diversification of the TPS-a genes. These findings imply that species undergoing rapid evolutionary changes, as indicated by their placement on long branches of the phylogenetic tree, may have experienced selective pressures favoring the expansion of their TPS-a gene repertoire. Such expansions could be adaptive, potentially enhancing the species’ ability to synthesize a diverse array of terpenoids, which are critical for various ecological functions including defense mechanisms and pollinator attraction. However, there were several exceptions. For instance, *L. chinense*, a magnoliid plant, contains 58 TPSs, including 24 TPS-a subgroup members. An expansion of the TPS-a subgroup is thought to have occurred after the split of monocots and dicots. The TPS-a subgroup mainly contains sesquiterpene synthases and is highly divergent in all seed plants [20]. We determined that the expansion of TPS-a occurred in monocots, super-rosids, and super-asterids after their split. The expansion of TPS-a in *L. chinense* appears to be unique compared to the other species, which is worth exploring. The significant expansion of the TPS-a subgroup likely reflects multiple biological mechanisms. First, ecological pressures such as co-evolution with specialized herbivores may drive selection for sesquiterpene diversity, as evidenced by TPS-a copy number variation across maize populations adapting to distinct pest pressures [49]. Second, genomic analyses in Arabidopsis reveal TPS-a clusters flanked by transposable elements, suggesting repeat-mediated duplication as a key expansion driver [50]. Finally, subfunctionalization through promoter divergence could enable tissue-specific expression partitioning of defense-related terpenoids [51]. These mechanisms collectively suggest that TPS-a expansions represent adaptive genomic responses to biotic challenges. The variable number of genes among taxa points to the independent loss or duplication of genes in different genomes. The number of TPSs in the TPS-b subgroup appears to be irregular and this subgroup has been lost in many Poaceae species (Figure 1). The lost functionalities due to the loss of TPS-b, such as mono-synthase activities, might be replaced by those of other subgroups. Notably, the absence of TPS-b raises questions about how Poaceae compensate for the loss of these volatile compounds. One possibility is that grasses have evolved alternative biosynthetic routes: TPS-g members might acquire monoterpene synthase activity through structural convergence, or non-TPS enzymes such as prenyltransferases could generate monoterpene precursors. Additionally, ecological shifts may reduce dependency on monoterpenes; the prevalence of wind pollination diminishes the need for floral volatiles, while silica-based physical defenses in grasses might offset the loss of monoterpene-mediated chemical protection.

Synteny could reflect important relationships between the genomic contexts of genes in terms of both function and regulation [52,53]. Therefore, analyzing the syntenic relationships of genes across a wide range of species provides important information about the evolution of gene families involved in plant growth and development. Phylogenomic synteny network analysis has been performed to examine the MADS-box, LEA (Late Embryogenesis Abundant), and MYB (v-myb avian myeloblastosis viral) gene families. Such analysis could be used to reveal genomic diversification, positional conservation, ancient tandem duplications, and lineage-specific transpositions [54,55,56]. However, this method did not appear to be so effective for analyzing the TPS gene family. First, among the 3802 TPSs of 115 angiosperms, only 1618s TPS were found to have syntenic relationships; this quantity is quite low. Second, in our syntenic cluster analysis, unlike the MADS-box and MYB gene families, many TPS edges could not be separated, and these edges were mixed and interwoven to form the huge synnet cluster126 (Tables S4 and S5). These features point to the complexity of TPS evolution. These TPSs without syntenic genes in other species represent “specialized gifts” prepared by angiosperms on their respective evolutionary journeys.

The ancestral TPS originated in land plants after their divergence from green algae and encoded a bifunctional copalyl/kaurene synthase (CPSKS) [8]. The TPS-c subgroup contains extant examples of bifunctional CPSKS, which are functionally analogous to the ancestral TPS required for the biosynthesis of gibberellins and related phytohormones [19,57]. As shown in Figure 2C, the syntenic relationships of TPS-c subfamily members are tightly connected. Moreover, phylogenetic analysis (Figure 3) indicated a high degree of conservation among TPS-c subfamily members in angiosperms. The TPS-e/f subgroup includes kaurene synthase genes, which are required for phytohormone biosynthesis. TPS-f is thought to be derived from TPS-e [22]. As shown in Figure 3, the TPS-e branch separated into two clades. These findings suggest that new functional domains may have appeared in the TPS-e subgroup of angiosperms.

Investigating TPSs is important for understanding the evolution of terpenoid biosynthesis in plants. Here, except for the important diterpenes (such as gibberellin and membrane sterols), we mainly focused on volatile monoterpenes and sesquiterpenes involved in floral scents. Rose is a great model in which to explore fragrance. We conducted a comprehensive analysis of the TPS gene family of *R. chinensis* (Figure 4 and Figure 5). Our data indicate that the expansion of TPS-a in *R. chinensis* mainly occurred via tandem duplication, and only one TPS-a subfamily member (RchiOBHmChr5g0037011) shares syntenic relationships with members in other angiosperms. The functions of the expanded TPS-a subfamily in rose (the types and contents of sesquiterpenes might also have expanded) are being intensively studied. While transcriptomic data suggest diverse expression patterns across RcTPS paralogs, our focused analysis of RcTPS23 demonstrates how phylogenomic synteny can prioritize candidates for functional studies. Systematic validation of additional TPSs remains an important direction for future research.

If non-syntenic TPSs expand, they may confer new terpene synthesis activities in plants. Analyzing TPSs with syntenic relationships might also shed light on the conservation of TPS function. Most terpenes are classified as secondary metabolites that help plants better adapt in their local environments [58]. Recent studies further elucidate TPS-mediated defense mechanisms across diverse species: In maize, ZmTPS12-produced dolabralexins confer resistance against *Fusarium graminearum* through direct antifungal activity [59]. Dendrobium orchids upregulate DcTPS7 expression under herbivore attack, catalyzing germacrene D synthesis to repel insects [60]. Potato StTPS2 synthesizes bulnesol/elemol diterpenes that systemically prime jasmonate signaling upon wounding [61]. Notably, tea plant CsTPS1 and CsTPS2 produce defense-related sesquiterpenes (δ-cadinene and α-humulene) that synergistically enhance resistance to *Ectropis obliqua* [62]. These findings collectively highlight the evolutionary convergence of TPS diversification in biotic stress adaptation. The sesquiterpene alcohol nerolidol and its derivative can induce the accumulation of defense-related compounds with extensive natural anti-herbivore or anti-pathogen effects [63,64]. Linalool, an acyclic monoterpene alcohol, is extremely widespread in plants. This compound attracts natural enemies of herbivores, thereby participating in the complex interplay between pollinator attraction and plant defense [65,66,67]. Using SynNet analysis, we detected the TPS-g subgroup member RcTPS23, which has dual functions in both nerolidol and linalool biosynthesis. RcTPS23 localizes to the cytosol (Figure 6 and Figure 7). A previous study of linalool nerolidol synthases in roses supports our results [68]. We speculate that this dual linalool nerolidol synthase activity is ubiquitous in angiosperms (Figure 6B). Although the subcellular localization of this bifunctional enzyme determines that it can only produce linalool or nerolidol in vivo (Figure 6A), both compounds can increase the ability of plants to resist biotic stress. Our findings lay the foundation for further exploring RcTPS23, and they increase our understanding of the phylogeny and functional differentiation of this important gene family in angiosperms. These insights directly inform rose breeding strategies. such as, overexpression of RcTPS23 in petal tissues could enhance floral fragrance (via linalool) while maintaining vegetative pest resistance (via nerolidol). The conserved TPS-g subgroup features identified here facilitate rapid ortholog discovery in hybrid rose cultivars for marker-assisted selection, etc.

## 4. Materials and Methods

### 4.1. Genomic Analysis of 115 Plant Species and the Genome-Wide Identification of TPSs

The publicly available genomes of 115 species were used for genome-wide identification of TPSs (Table S1). These 115 angiosperms include 21 super-asterids, 61 super-rosids, five basal-eudicots, 26 monocots, one magnoliids (*L. chinense*), and one basal-angiosperm (*A. trichopoda*). The download websites of whole-genome protein sequences this study used can be obtained in Table S1. The R package (version 0.6.5) plant list was used to check the species status [69]. The phylogenetic relationships were analyzed with Phylomatic (version 3), and the Figtree program (version 1.4.4) was used to visualize the phylogenetic tree. All-against-all comparisons between pairwise genomes and the detection of synteny blocks were performed using a GitHub script (https://github.com/zhaotao1987/SynNet-Pipeline) (accessed on 1 December 2021).

Candidate terpene synthase (TPS) genes were identified using the Hmmsearch program in the HMMER3.0 package [70] with default settings and were confirmed using the Pfam database. All TPSs were identified based on the presence of two specific domains: the Pfam N-terminal domain (PF01397) and the Pfam C-terminal domain (PF03936) [20]. Significant hits (*E*–value < 10^−3^) were used to identify candidate TPSs that were encoded in the genomes of 115 angiosperms (nodes) (Table S2). The protein sequences of identified TPSs have been listed in the FASTA file “TPSs.fasta”.

### 4.2. Subgroup Classification and Phylogenetic Analysis of TPSs

After domain verification, the 3802 TPS protein sequences were classified into six TPS subgroups. Based on the method of [20], TPS-e and TPS-f were combined into one subgroup: TPS-e/f. Multiple amino acid sequence alignment was performed using ClustalX (version 2.0) with standard parameters. The results were analyzed using the online program MEME (http://meme-suite.org/, (accessed on 30 January 2022)), and the alignments were manually adjusted to classify the conserved motifs of TPSs, such as ‘RRX_8_W’, ‘DDxxD’, ‘DxDD’, and ‘NSE/DTE’. The multiple sequence alignment results were saved in fasta format.

A phylogenetic tree of the chosen TPS protein sequences was constructed using RAxML [71] with the maximum-likelihood method based on the best substitution model Jones-Taylor-Thornton (JTT) [72], with 1000 bootstrap replicates. The phylogenetic tree was visualized using the Figtree program (version 1.4.4) and iTOL online software (version 5) [73].

### 4.3. Synteny Block Detection, Network Construction, and Network Clustering of TPSs

The SynNet pipeline described by Zhao [37] was used for syntenic block calculations, network construction, and analysis of cluster distribution (https://github.com/zhaotao1987/SynNet-Pipeline) (accessed on 1 December 2021). The synteny blocks in the 115 species were identified with MCScanX software (https://github.com/wyp1125/MCScanX) (accessed on 20 December 2021) [74] using default parameters (minimum gene number in a collinear block = 5, maximum gaps = 25). The output files of TPS genes syntenic blocks (edges, with headers Locus_1 and Locus_2) are provided in Tables S3 and S4. A synteny network (SynNet) of syntenic blocks was built containing all the TPS genes in the 115 species, and the SynNet was imported and visualized in Gephi (version 0.9.1) [75].

The clique percolation method in CFinder was used to locate all putative clique communities (k = 3) in the TPS gene synteny network (synnets) [76,77]. The synnets were used to identify communities (clusters of gene nodes) (Tables S4 and S5), and these synteny communities were further used for phylogenetic profiling. For each genome, the number of syntenic gene copies of each cluster was counted, as shown in Table S6. The Jaccard method of the vegan package [78] was carried out to calculate the dissimilarity index of all clusters, followed by separate hierarchical clustering and visualization of these clusters using “ward. D” and “pheatmap”.

### 4.4. Chromosomal Localization and Intron-Exon Structure Analysis of RcTPSs

Information about chromosomal locations and gene structures was obtained from the gff3 file in the *R. chinensis* genome database (RchiOBHm-V2) [33] and is provided in Table S7. Mapchart [79] was used to visualize the chromosomal locations of all putative functional terpene synthases in *R. chinensis.* The online software GSDS (http://gsds.gao-lab.org/, (accessed on 15 January 2022)) was used to depict the introns and exons in the 54 *RcTPS* genes.

### 4.5. Gene Expression Analysis of RcTPSs

Two RNA-seq data sets were used to obtain rose TPS gene expression data by the TPM method (transcripts per kilobase million) [80]. One data set includes transcriptome data from rose petals at different stages of development (BioProject PRJNA351281, Table S8, Q30 ≥ 92%, mapping rate ≥ 85%, Error rate ≤ 0.02), and the other contains transcriptome data from different rose tissues (root, stem, leaf, prickle, stamen, pistil, and ovary tissue) (BioProject PRJNA546486, Table S9, Q30 ≥ 93%, mapping rate ≥ 90%, Error rate ≤ 0.03). Heat maps representing gene expression levels were drawn with the R package pheatmap (version 1.0.12).

### 4.6. Reagents and Materials

Commercial reagents included: Plant RNA Purification Reagent (Promega, Madison, WI, USA, Cat# A7280); PrimeScript RT Reagent Kit with gDNA Eraser and SYBR Premix Ex Taq (Takara Bio, Shiga, Otsu, Japan, Cat# RR047A/RR420A); KOD-Plus-Neo DNA Polymerase (TOYOBO, Osaka, Japan, Cat# KOD-401); KpnI/XhoI restriction enzymes (New England Biolabs, Ipswich, MA, USA, Cat# R3142/R0146); In-Fusion HD Cloning Kit (Takara Bio, Cat# 639649); Ni Sepharose 6 Fast Flow (GE Healthcare, Chicago, IL, USA, Cat# 17531801); isopropyl-β-D-thiogalactopyranoside (IPTG, Sigma-Aldrich, St. Louis, MO, USA, Cat# 367-93-1); farnesyl diphosphate (FPP, Cat# 44270) and geranyl diphosphate (GPP, Cat# G6772) from Sigma-Aldrich. Instrumentation comprised: CFX96™ Real-Time PCR system (Bio-Rad Laboratories, Hercules, CA, USA); Leica TCS SP8 confocal microscope (Leica Microsystems, Wetzlar, Germany); Agilent 7890A/5975C GC-MS system with HB-5MS column (5% phenyl methyl silox, Agilent J&W Scientific, Folsom, CA, USA). Oligonucleotide primers were synthesized by Sunbiotech Co., Ltd. (Beijing, China). General chemical reagents were purchased from Sangon Biotech (Shanghai, China).

### 4.7. Validation of Gene Expression Data by qRT-PCR

Quantitative reverse-transcription PCR (qRT-PCR) was performed to validate the gene expression levels of *RcTPS23* in eight tissues of *R. chinensis* ‘Old Blush’ (petals at four stages: FB_GP, FB_CP, FB_PP, OF_PP, stamen, leaf, stem, and root tissues). A Plant RNA Isolation Kit was used to extract total RNA from each sample. A PrimeScript RT Reagent Kit with gDNA Eraser was used to synthesize first-strand cDNA from 1.0 µg total RNA. The reaction mixture contained 10 µL SYBR Premix Ex Taq, 0.4 µL 10 µM forward and reverse transcript-specific primers (forward primer: 5′-TTTAGAGAAGCAATACACCAGGTC-3′, reverse primer: 5′-CAGGGAAGCCTTGTTGTCTTA-3′), 2 µL cDNA, and 7.2 µL sterile distilled water. The qRT-PCR was performed using a CFX96™ Real-Time PCR system with the following program: 95 °C for 30 s; 40 cycles of 95 °C for 5 s and 60 °C for 30 s; and a final melting curve analysis of 95 °C for 15 s, 60 °C for 1 min, and 95 °C for 15 s. The relative gene expression levels were normalized against the expression level of the endogenous reference gene *RcActin* [81] and calculated using the 2^−ΔΔCt^ method [82]. Each sample was examined with three biological replicates. The histograms were generated using the Origin9 program (OriginLab, Northampton, MA, USA). All commercial reagents and kits used in this section are detailed in Section 4.6 (Reagents and Materials).

### 4.8. Subcellular Localization

The cDNA sequence of *RcTPS23* was amplified using the forward primer 5′-GGTACCATGACGTTCATTCTTCAAGC-3′ and the reverse primer 5′-CTCGAGGCTAGCCATGAGTGATGC-3′ under the following conditions: 94 °C for 2 min, followed by 35 cycles of 94 °C for 10 s, 54 °C for 15 s, and 72 °C for 2 min, with a final extension of 72 °C for 5 min, using KOD-Plus-Neo DNA Polymerase. The PCR product was cloned into the pEZS-NL vector, which had been cleaved with KpnI/XhoI. *A. thaliana* protoplast isolation and transformation were carried out according to a standard protocol [83]. Using empty vector-transfected protoplasts as the control. Reagent sources are provided in Section 4.6. The transformed protoplasts were incubated at 23 °C for 16 h and observed under a laser confocal microscope (Leica TCS SP8, Wetzlar, Germany) to detect eGFP fluorescence; eGFP was excited at 488 nm.

### 4.9. Generation of Recombinant RcTPS23 and In Vitro Enzyme Activity Assays

The cDNA sequence of *RcTPS23* was cloned into the *pET-32a* vector by In-Fusion cloning using the forward primer 5′-GGCTGATATCGGATCCATGACGTTCATTCTTCAAGC-3′ and the reverse primer 5′-CAAGCTTGTCGACGGAGCTCGCTAGCCATGAGTGATGC-3′ and transformed into *E. coli* strain BL21 (DE3). Recombinant protein production was induced by adding 0.8 mM isopropyl-b-d-thiogalactopyranoside (IPTG) and incubating at 16 °C for 24 h. The *E. coli* cells were sonicated and centrifuged to obtain the supernatant, and proteins were purification on a Ni Sepharose column. The purified proteins were collected and concentrated.

In vitro enzyme activity assays of RcTPS23 recombinant protein were carried out using 50 μg purified protein in 200 μL of assay buffer [50 mM HEPES (pH 7.4), 25 mM MgCl_2_, 5 mM DTT, 5% (*v*/*v*) glycerol, 50 μM FPP or 50 μM GPP]. The blank control contained the same buffer and substrates but no recombinant protein. The samples were incubated at 30 °C for 2 h, and the volatile products were absorbed on 100 μm PDMS fiber prior to GC-MS analysis. The PDMS fiber was transferred to the injection port (250 °C) of the GC-MS system and desorbed for 5 min. GC was performed using a GC system coupled with a mass spectrometer. For GC, an HB-5MS column (5% phenyl methyl silox: 30 m × 250 µm i.d., 0.25 µm) was used for all samples. The analytical conditions were as follows: The initial temperature was held at 50 °C for 1 min and raised to 210 °C for 2 min at 3 °C/min, maintained for 3 min, and increased to 230 °C at 15 °C/min. The analysis was conducted in split-less mode; helium was used as the carrier gas at a rate of 1.0 mL/min. The mass spectrometer was set as follows: 230 °C, electron-impact (EI) model with 70 eV electron power; 280 °C for the auxiliary temperature. The scan range was 80–500 *m*/*z*. Refer to Section 4.3 for reagent specifications. The volatile products were identified and characterized by comparing mass spectra using AMDIS software (Automated Mass Spectral Deconvolution and Identification System, http://www.amdis.net/, (accessed on 10 May 2022)) and the NIST Mass Spectral Library (https://www.nist.gov/, (accessed on 10 May 2022)). The assay conditions were established based on previously validated methods for terpene synthase characterization [84,85].

## 5. Conclusions

Our research across 115 angiosperms reveals the genomic diversity and evolutionary trajectories of the terpene synthase (TPS) gene family, identifying 3802 TPS genes and categorizing them into five distinct subgroups. This extensive classification highlights significant variability in gene numbers, with adaptive expansion observed in the TPS-a subgroup. Synteny analysis further delineated homologous relationships and evolutionary conservation, identifying 22,035 edges and 1618 nodes that map the genetic architecture and evolutionary pressures shaping these genes. Notably, lineage-specific expansions linked to whole-genome duplications suggest significant evolutionary roles. Our focused study on *R. chinensis* uncovered a large TPS gene family with a substantial expansion in the TPS-a subgroup. This study enhances our understanding of plant secondary metabolism and sets a foundation for future explorations into the ecological and adaptive significance of TPS genes. It should be acknowledged that our study has certain limitations: the angiosperm-centric sampling may underrepresent early plant lineages, functional validation was limited to RcTPS23, and ecological correlations remain unexplored. These findings hold biotechnological promise for fragrance compound biosynthesis (e.g., engineering TPS-a genes like RcTPS23 for industrial terpene production) and agricultural innovation (e.g., developing pest-resistant crops through volatile organic compound engineering). Future investigations should prioritize integrating multi-omics approaches to link genomic patterns with terpene metabolite profiles, and employing high-throughput CRISPR screening to decipher genotype–phenotype relationships across plant taxa.

## Figures and Tables

**Table 1:** Taxonomy of plant TPS subgroups.

Subgroup	Classification	Domain Composition	Motif Features	Distribution	Subcellular Localization	Type of Products
TPS-a	Class I	βα	With ‘RRX_8_W’ motif (the second ‘R’ is not conserved)	Angiosperms	M/P/C	Sesqui-TPSs
TPS-b	Class I	βα	With conserved ‘RRX_8_W’ motif	Angiosperms	P/C	Mono-TPSs, Isoprene synthase
TPS-g	Class I	βα	Without ‘RRX_8_W’ motif	Angiosperms	P/C	Acyclic mono-, Sesqui- and Di-TPSs
TPS-h	Class I/II	γβα	Contain both ‘DxDD’ and ‘DDxxD’ motifs	Mosses, Liverworts, Lycophytes and Ferns	unknown	Di-TPSs
TPS-e/f	Class I	γβα/βα	Without ‘DxDD’ motif	Vascular plants	M/P/C	Ent-kaurene synthase (KS); Mono-, Sesqui-, and Di-TPSs
TPS-c	Class II	γβα/βα	With conserved ‘DxDD’ motif	Land plants	M/P	Copalyl diphosphate synthase (CPS), CPS/KS; Mono-, Sesqui-, and Di-TPSs
TPS-d	Class I/II	βα/γβα	Contain both ‘DxDD’ and ‘DDxxD’ motifs	Gymnosperms	unknown	Mono-, Sesqui- and Di-TPSs

Abbreviation: Di-, diterpene; Mono-, monoterpene; Sesqui-, sesquiterpene; C, cytosolic; P, plastidic; M, mitochondrial.

## Data Availability

The data are contained within the article and Supplementary Materials.

## Supplementary Materials

The following supporting information can be downloaded at: https://www.mdpi.com/article/10.3390/ijms26052113/s1.
