# Supplementary material for: Phylogeny and Functional Differentiation of the Terpene Synthase Gene Family in Angiosperms with Emphasis on Rosa chinensis
Source: Int J Mol Sci. 2025 Feb 27;26(5):2113. doi: 10.3390/ijms26052113 (PMC11901113; doi:10.3390/ijms26052113)
Supplement: Supplementary file 1 [file ijms-26-02113-s001.zip › Supplementary materials.pdf]

**Supplementary materials**

**Figure S1.** Phylogenetic profiling of all putative clique communities (k = 3). The phylogenetic profile shows the number and distribution of TPSs in 115 species. Each species is color-coded to represent different plant groups: super-asterids (light blue), super-rosids (light orange), basal-eudicots (light purple), and monocots (light green), with *L. chinense* and *A. trichopoda* highlighted in light gray. Red and blue asterisks indicate events of whole-genome duplication and whole-genome triplication, respectively. A color scale is used to depict the number of nodes per cell.

**Figure S2.** Phylogenetic analysis of subgroups TPS-c and -e/f in 115 angiosperm species (including node ids). This phylogenetic tree constructed from the TPS-c and TPS-e/f gene members in 115 angiosperms, detailed in Table S1. The tree segregates into clades that are color-marked to represent various plant categories: super-rosids (light orange), monocots (light green), super-asterids (light blue), basal eudicots (light purple), and basal angiosperms and magnoliids (light gray).

**Figure S3.** Exon-intron structure and phylogenetic relationships of RcTPS genes. Exons are depicted as solid black boxes; black lines indicate introns. The maximum-likelihood phylogenetic tree was constructed with 1,000 bootstrap replicates.

**Table S1.** The information of 115 plant genomes which be analyzed.

**Table S2.** Total node information of TPSs in 115 species

**Table S3.** Total edges information of TPSs in 115 species.

**Table S4.** The node list of 127 characterized clusters. (k = 3)

**Table S5.** The edge list of 127 characterized clusters. (k = 3)

**Table S6.** Number of syntenic genes and syntenic clusters of TPSs in 115 species.

**Table S7.** The information of physical position of the 54 *RcTPSs* in the 7 chromosome scaffolds of *R. chinensis*.

**Table S8.** The TPM of *RcTPSs* in four stages petal of *R. chinensis*.

**Table S9.** The TPM of *RcTPSs* in different organs of *R. chinensis*.
**TPSs.fasta** The protein sequences of TPSs in 115 angiosperm species.
