# Supplementary figures and images for: Phylogeny and Functional Differentiation of the Terpene Synthase Gene Family in Angiosperms with Emphasis on Rosa chinensis
Source: Int J Mol Sci. 2025 Feb 27;26(5):2113. doi: 10.3390/ijms26052113 (PMC11901113; doi:10.3390/ijms26052113)

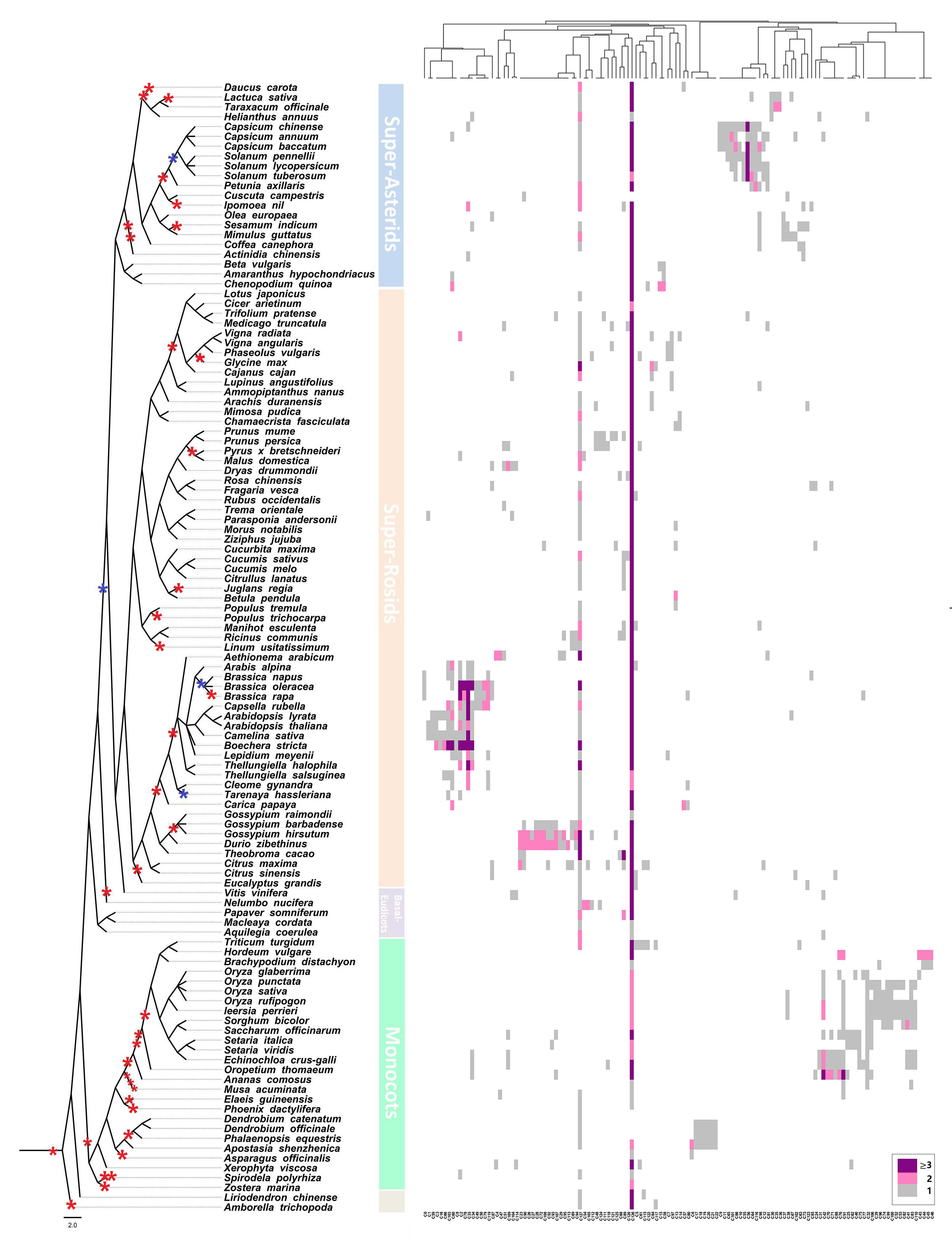

Supplement: Supplementary file 1 [file ijms-26-02113-s001.zip › Figure S1.tif]

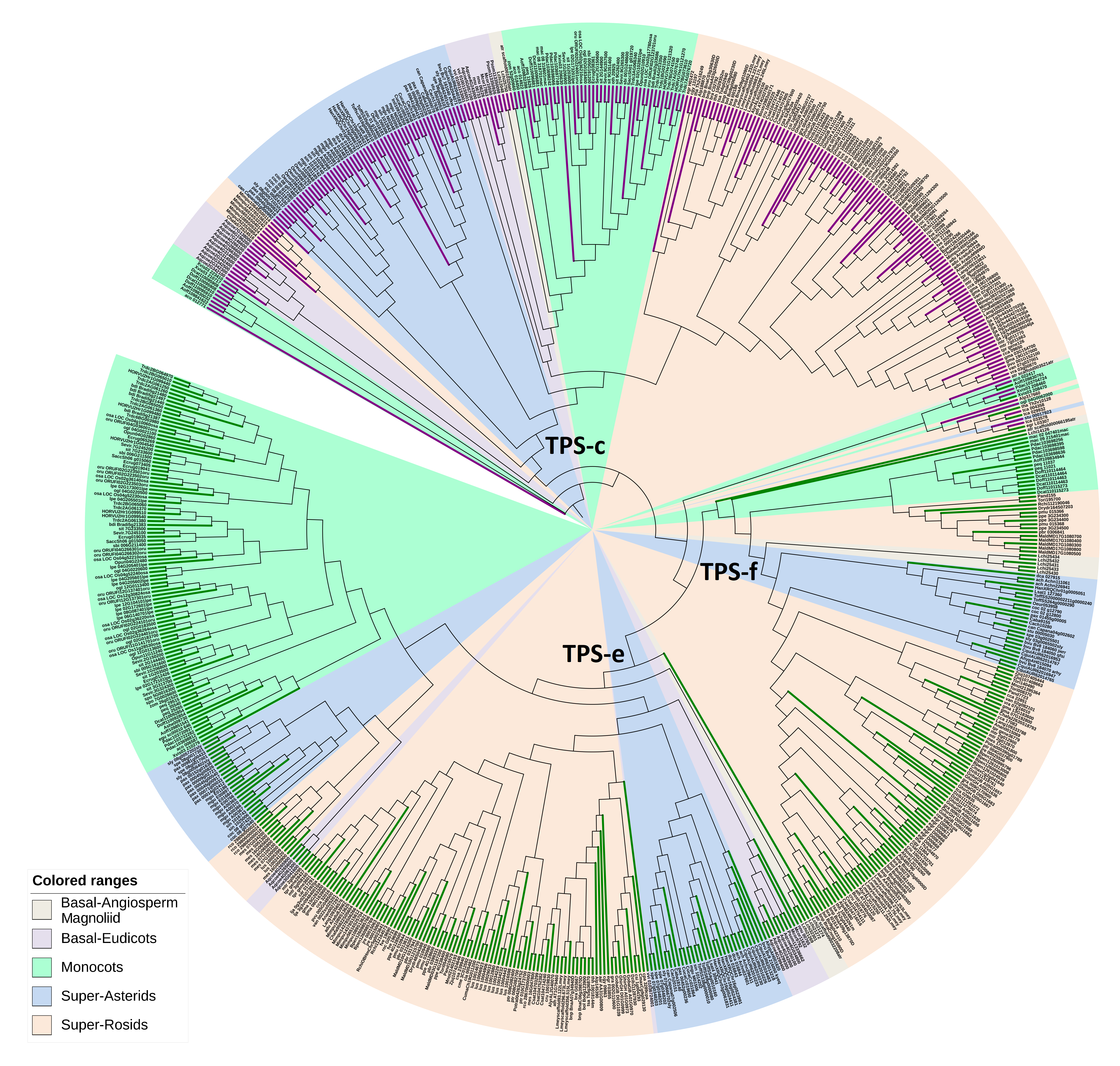

Supplement: Supplementary file 1 [file ijms-26-02113-s001.zip › Figure S2.tif]

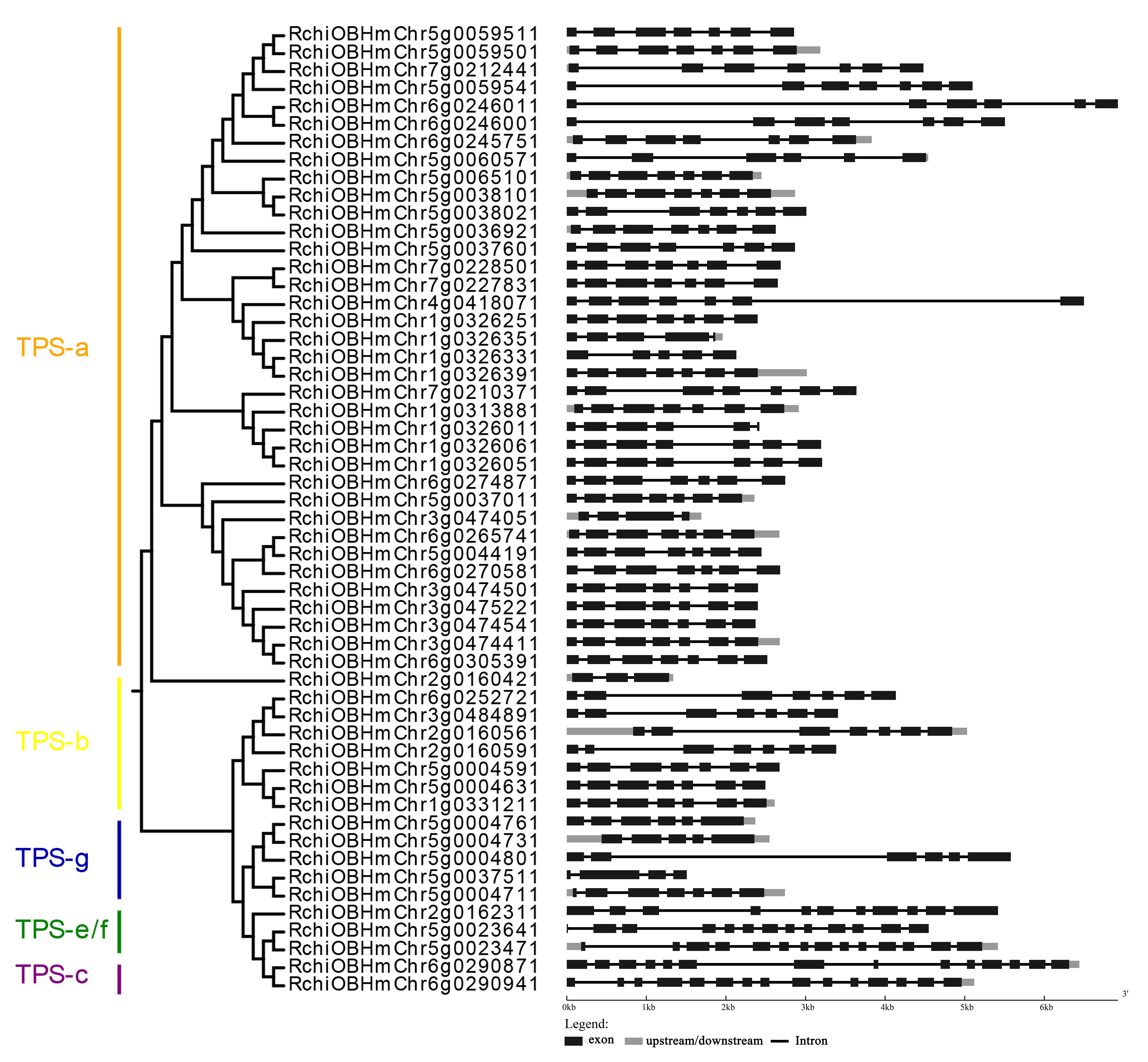

Supplement: Supplementary file 1 [file ijms-26-02113-s001.zip › Figure S3.tif]
